# Screen violence: a real threat to mental health in children and adolescents

**DOI:** 10.1016/j.lana.2023.100473

**Published:** 2023-03-09

**Authors:** The Lancet Regional Health – Americas

Screen violence has been defined as “depictions of characters (or players) trying to physically harm other characters (or players)”. It is as old as the origin of screen media itself, with very violent scenes included in the first multi-reel film “The Story of the Kelly Gang”, screened in Melbourne in 1906. Evidence suggests that watching violent TV programmes and engaging with violent video games are associated with aggressive behaviour in children, teenagers, and young adults, both in the short and long term. Although the association between screen violence and aggressive behaviour appears to be modest, this suggests that other factors also play a part in the development of aggression.

Screen violence is globally widespread and has become easily accessible and available on demand in the last decade, courtesy of technology (such as cable TV, tablets, smart phones, and social media platforms). Not even poverty, probably except for extreme poverty, seems to protect many people from being exposed to screen violence because even the simplest TV can be found in modest homes and can be the only source of entertainment in remote areas. Social media platforms (such as YouTube, TikTok, Facebook, Twitter, WhatsApp, and Instagram) are extremely popular among adults and children. With an estimated 6.8 billion people worldwide owning a smartphone, nearly 87% of the world's population, immediate access to violent content has increased exponentially.

Exposure to violent content can decrease empathy and cause increased aggressive thoughts, anger, and aggressive behaviour. A meta-analysis of 24 studies from Canada, the USA, Germany, Japan, the Netherlands, and Singapore concluded that engaging with violent video games was related to aggression. This study confirms earlier findings from another meta-analysis of 130 research reports, comprising 130,000 participants. Time spent watching screen violence has also been directly associated with increased bullying and cyberbullying in both boys and girls. In addition, aggressive behaviour during childhood appears to be an important predictor of violent behaviour in older adolescents and young adults.

In the 1970s, the TV industry in the USA put pressure on the US Government to exclude prominent researchers in psychology and aggressive behaviour from a committee—that was set to study the effects of TV violence on behaviour and mental health. This resembles one of the strategies traditionally used by the tobacco companies to oppose unfriendly health policies: to distort science. Certainly, there is a large amount of money at stake. The global revenue value in TV & video segment, video games, and broadband internet services combined is expected to grow to about US$1300 billion in 2023, by far more than the global tobacco market value.

Despite the negative impact of screen violence on behaviour, little has been done to reduce screen violence, particularly in children and adolescents. Watersheds on TV content and ratings for TV programmes and movies are some of the most common policies implemented to regulate screen media in many countries, including those in Latin America. Yet many PG-13-rated programmes are full of violence. Moreover, these policies do little or nothing to address screen violence available on the internet, including social media platforms and video games. In some countries, policies and recommendations focus primarily on parental responsibility, which is of little help if no adult is present. For example, according to the National Television Council in Chile, 52% of children aged 6–12 years watch audio-visual content without adult supervision. This suggests that parental control might not be enough to tackle this highly complex problem that could require interventions at multiple levels. It is likely that screen violence will be more difficult to deal with than other trade issues, such as tobacco and alcohol, because screen content can reach continents within seconds, with virtually no barriers or time to filter content.

More research is needed to better understand the impact of screen violence on mental health. For instance, a topic that has been little explored is the possible effect of violence displayed in social media platforms on aggressive behaviour. This information would be very valuable given the exponential growth of popularity of these internet platforms, which have little regulation. Further research is also required to investigate whether there is an association between chronic exposure to screen violence and addiction, crime, suicide, or mass shooting.

In the latest State of the Union, on February 7, Joseph Biden Jr (president of the USA) addressed the need to do more for children's mental health and to regulate social media companies and their impact on children. “We must finally hold social media companies accountable for the experiment they are running on our children for profit,” Biden said. Policymakers and governments should put screen violence in their agenda to tackle this real threat to mental health, re-assess the growing scientific evidence, implement stricter policies, and educate children and adolescents so that they know how to discriminate and process the content of visual media. As many things in life, screen media has wonderful positive aspects, but can also have serious consequences on health if not used and regulated properly. Ethical considerations should help set limits of what is reasonably permissive for screen content, particularly for children and adolescents. Further action is urgently needed to regulate violent content in screen media.

